# Effects of concurrent chemoradiotherapy with or without Endostar on the regression of retropharyngeal lymph node and prognosis of patients with locally advanced nasopharyngeal carcinoma: a retrospective study

**DOI:** 10.1007/s00432-024-05762-x

**Published:** 2024-05-04

**Authors:** Jun-Mei Song, Ning Mo, Yu-Qing Lv, Lu-Lu Huang, Ya-Jing Wen, Ting Liu, Zhi-Ru Li, Ren-Sheng Wang, Ting-Ting Zhang

**Affiliations:** 1 Department of Radiation Oncology, The First Affiliated Hospital of Guangxi Medical University, Nanning, 530021 Guangxi China; 2 Oncology Department, Nanchong Central Hospital, The Second Clinical Institute of North Sichuan Medical College, Nanchong, 637000 Sichuan China; 3 Laboratory of Early Prevention and Treatment for Regional High Frequency Tumor (Guangxi Medical University), Ministry of Education, Nanning, 530021 China; 4 Department of Radiation Oncology, Nanfang Hospital, Southern Medical University, Guangzhou, 510515 China

**Keywords:** Locally advanced nasopharyngeal carcinoma, Endostar, Retropharyngeal lymph nodes, Prognosis

## Abstract

**Background and Purpose:**

To investigate the effect of combining Endostar with concurrent chemoradiotherapy (ECCRT) compared to concurrent chemoradiotherapy (CCRT) on the regression rate of retropharyngeal lymph nodes (RLNs) and the relationship between regression rate of RLNs and prognosis of patients with locally advanced nasopharyngeal carcinoma (LANPC).

**Methods:**

A total of 122 LANPC patients with RLNs metastasis were included. Metastatic RLNs were delineated both before and after treatment slice by slice on the magnetic resonance images cross-section. The regression rate of RLNs, adverse effects (AE) were evaluated. The median regression rate of RLNs was taken as the cut-off value, and the patients were furtherly divided into high regression rate (HRR) group and low regression rate (LRR) group, then survival times were evaluated.

**Results:**

The median regression rates of RLNs in the ECCRT and CCRT groups were 81% and 50%, respectively (P < 0.001). There was no statistically significant difference in the incidence of grade 3/4 AEs between the two groups, except for oral mucositis (ECCRT 26.23% *vs.* CCRT 44.26%, P = 0.037). The 3-year overall survival (OS), progression-free survival (PFS), distant metastasis-free survival (DMFS) and locoregional failure-free survival (LRFFS) rates in the HRR and LRR groups were 85.48% and 86.67% (P = 0.983), 80.65% and 68.33% (P = 0.037), 83.87% and 85% (P = 0.704), 93.55% and 81.67% (P = 0.033), respectively.

**Conclusions:**

Patients in the ECCRT group had higher regression rates of RLNs and lower incidence of severe oral mucositis. Furthermore, patients in the HRR group had a better 3-year PFS and LRFFS rate than those in the LRR group.

## Introduction

Nasopharyngeal carcinoma (NPC) has a high prevalence in southern China and is often accompanied by lymph node metastasis. A considerable portion (ranging from 66.2% to 86.3%) of NPC patients were initially diagnosed with metastatic retropharyngeal lymph nodes (RLNs) (Huang et al. 2019; Chen et al. 2022). The RLNs and the level II lymph nodes seem to be the first lymph nodes of nasopharyngeal carcinoma metastasis (Liu et al. 2006; Ho et al. 2012; Wang et al. 2015). The presence of RLNs metastasis in NPC patients was recognized as a main negative prognostic factor (Coskun et al. 2011; Ma et al. 2007). Regional failure has been reported in 6.2–7.7% of patients with NPC after treatment (Kim et al. 2022; Li et al. 2017; Xue et al. 2017). Among NPC patients with local recurrence, 43.8–52.9% of recurrent lesions were located in the retropharyngeal region (Kim et al. 2022; Xue et al. 2017).

According to the guidelines, concurrent chemoradiotherapy (CCRT) is the standard treatment option for stage II-IVa NPC (Chen et al. 2021a, b). However, 28.2% of patients still have residual RLNs after three months of intensity-modulated radiotherapy (IMRT) (Li et al. 2020a, b). Moreover, several researches showed that about 50% of patients with non-metastatic NPC had abnormally enlarged RLNs after radical treatment (Meng et al. 2020; Tan et al. 2023). Meanwhile, residual RLN was an unfavorable prognostic factor for overall survival (OS), progression-free survival (PFS), distant metastasis-free survival (DMFS), and locoregional failure-free survival (LRFFS) (Li et al. 2020a, b). However, only a few studies reported the prognostic value of regression rate of RLN in locally advanced nasopharyngeal carcinoma (LANPC). Therefore, in-depth exploration of the prognostic value of RLNs in LANPC may have positive clinical significance.

The initiation of angiogenesis is important in the growth and metastasis of the tumor cells (Rankin et al. 2016). It is known that vascular endothelial growth factor (VEGF) is an inducer that promotes angiogenesis and tumor progression (Tan et al. 2017). Endostar (recombinant human endostatin injection), a targeted drug against VEGF receptors, has been found to not only inhibit tumor angiogenesis, but also suppress the generation of tumor lymphatic vessels and lymphatic metastasis (Shang et al. 2014). Previous study had indicated that IMRT combined with Endostar in the treatment of LANPC had better efficacy and fewer serious adverse effects than CCRT (Chen et al. 2021a, b). However, the effect of Endostar on the regression of RLNs remains to be explored, and the evaluation of RLNs was one-dimensional in the past. Here, we compared the regression of RLNs using three-dimensional quantitative measurement in LANPC patients who received Endostar combined with CCRT and CCRT alone.

## Materials and methods

### Patients

Our study retrospectively analyzed the clinical data of LANPC patients with RLNs metastasis who were treated at the First Affiliated Hospital of Guangxi Medical University from January 1, 2015 to April 30, 2020. Criteria for eligibility are as follows: (1) newly pathologically diagnosed and untreated stage III-IVa (based on the Union for International Cancer Control /American Joint Committee on Cancer 8th edition staging system) NPC patients; (2) aged 18–75 years; (3) RLN metastasis; (4) complete clinical data; (5) magnetic resonance imaging (MRI) of the head and neck performed before and after the radiotherapy; (6) no previous or concurrent malignancies; (7) normal hematologic, liver, renal and heart functions; (8) Eastern Cooperative Oncology Group score 0–1. The present study was approved by the medical ethics committee of the First Affiliated Hospital of Guangxi Medical University.

### Treatment

The target delineation followed the international guideline for the delineation of the clinical target volumes (CTV) for NPC (Lee et al. 2018), and radiotherapy process was under the guidance of Report 50 and Report 62 of International Commission on Radiation Units and Measurements. 68–74 Gy was as the prescription dose for PGTVnx; 66–70 Gy was for PGTVnd; 60–66 Gy was for PCTV1; and 50–56 Gy was for PCTV2 (5 fractions per week for 30–33 fractions). Chemotherapy regimens were based on platinum (80–100 mg/m^2^, every 3 weeks or 40mg/m^2^, every week). Endostar (7.5 mg/m^2^/day, day 1–10, every 3 weeks) was continuously pumped intravenously from 5 days before radiotherapy according to the previous study (Yin et al. 2022).

### Evaluation and volume measurement of retropharyngeal lymph nodes

Images were acquired using a 1.5-T MRI scanner (GE Healthcare Life Sciences, Little Chalfont, UK). All patients underwent both routine and enhanced scans including nasopharynx and neck. RLNs, which fused with primary tumors, were clearly distinguished by either a contrast-enhancing rim or a disparity in signal intensity when compared to the primary tumor (Liu et al. 2006). The diagnosis of positive RLNs met the criteria proposed by Head and Neck Cancer Radiotherapy Atlas (Luo 2020). And any RLNs with the maximum standardized uptake value (SUVmax) > 4.5 by F-18 fluorodeoxyglucose (FDG) positron emission tomography (PET)/computed tomography (CT) (Matsubara et al. 2012).

The image data from T2 plain-scan weighted axial MRI were imported into the Varian Eclipse radiation treatment planning system in the DICOM format. Then the positive and residual RLNs were delineated slice by slice. The contouring was verified by two medical professionals (a trained radiation oncologist and a radiological expert) following the principle of consensus. If there was disagreement between them, further discussions were held by an expert team composed of two radiation oncology specialists and one chief radiologist. The Eclipse system is used to automatically calculate the RLNs’ volume accurately. The RLNs volume before and after radiotherapy is defined as RNV_before_ and RNV_after_, respectively. The RNVs regression rate = (RNV_before_ -RNV_after_) /RNV_before_* 100% (Lee et al. 2016; Li et al. 2023). The median regression rate of RLNs was taken as the cut-off value, and the patients were divided into high regression rate (HRR) and low regression rate (LRR).

### Adverse effects (AEs) evaluation

Acute AEs were evaluated from the beginning to 90 days after radiotherapy following the National Cancer Institute Common Terminology Criteria v4.0. Late AEs occurred later than 90 days after radiotherapy. Radiation Therapy Oncology Group and European Organization for Research and Treatment of Cancer standards were used to assess early and late radiation side effects.

### Follow-up

Follow-up duration was determined from the date of pathologically diagnosed until either the date of the last medical encounter date or death. Patients underwent examinations every 3 months for the 0–2 years after treatment; every 6 months within the 3–5 years, and every year beyond 5 years. Post-treatment visits comprised physical examination, fiberoptic nasopharyngoscopy, MRI/CT of the head and neck, hematology and biochemistry examinations, chest CT, and abdominal ultrasound/CT. When patients experienced bone pain, an emission computed tomography for bones should be performed.

### Statistical methods

SPSS 25.0 statistical software (IBM Corp., Armonk, NY, USA) and GraphPad Prism 8.0 were used. Non-normal continuous variables were described using median and interquartile range, while categorical variables were described in percentages. Statistical comparisons between groups were conducted using the rank-sum test, and chi-square test/Fisher precision method. The OS, PFS, DMFS, and LRFFS were calculated with the Kaplan–Meier method. Then the log-rank test was employed for comparison. The Cox proportional hazards model was employed to analyze the hazard ratio (HR), and to determine the corresponding 95% confidence intervals (CIs). Gender, clinical stage, age, pathological types, ECOG score, smoking, drinking, laterality, necrosis, ENS, T stage, N stage and regression rate of RLNs were included in the univariate analysis. Covariates with P ≤ 0.20 in univariable analysis were included as covariates in multivariable analysis. All statistical tests were bilateral with a significance level set to P < 0.05.

## Results

### Patient characteristics

A total of 122 LANPC patients were enrolled with 61 patients in each group. The detailed characteristics of patients are shown in Table 1. All 122 patients completed the entire radiation therapy process and all the patients in the ECCRT group completed 3 cycles of Endostar. In the ECCRT group, 6 patients (9.84%) experienced a 25% reduction of chemotherapy drug dosage due to serious AEs; while in the CCRT group, 4 patients (6.56%) also underwent a reduction.

**Table 1:** Clinical characteristics in the ECCRT and CCRT group

Characteristics	ECCRT n = 61	CCRT n = 61	P
*Age*			0.769
≥ 60	7 (11.48%)	6 (9.84%)	
< 60	54 (88.52%)	55 (90.16%)	
*Gender*			0.410
Male	43 (70.49%)	47 (77.05%)	
Female	18 (29.51%)	14 (22.95%)	
Clinical stage*			0.587
III	31 (50.82%)	28 (45.90%)	
IVa	30 (49.18%)	33 (54.10%)	
*Pathological types*			1.000
WHO II	4 (6.56%)	5 (8.20%)	
WHO III	57 (93.44%)	56 (91.80%)	
*ECOG score*			0.364
0	35 (57.38%)	30 (49.18%)	
1	26 (42.62%)	31 (50.82%)	
*Smoking*			0.258
Yes	19 (31.15%)	25 (40.98%)	
No	42 (68.85%)	36 (59.02%)	
*Drinking*			0.533
Yes	14 (22.95%)	17 (27.87%)	
No	47 (77.05%)	44 (72.13%)	
*Chemotherapy cycles*			1.000
≥ 2	58 (95.08%)	57 (93.44%)	
1	3 (4.92%)	4 (6.56%)	
*T stage*			0.481
T1-2	2 (3.28%)	7 (11.48%)	
T3	35 (57.38%)	31 (50.82%)	
T4	24 (39.34%)	23 (37.70%)	
*N stage*			0.094
N0-1	19 (31.15%)	12 (19.67%)	
N2	34 (55.74%)	36 (59.02%)	
N3	8 (13.12%)	13 (21.31%)	

*TNM staging followed the 8th edition of the American Joint Commission on Cancer (AJCC) staging system

WHO, World Health Organization; ECOG, Eastern Cooperative Oncology Group

### RLNs

The comparable baseline features of the RLNs before radiotherapy were presented in Table 2 (P > 0.05). The median volume of RLNs in the ECCRT and CCRT groups before treatment were 3.90 cm^3^ and 4.81 cm^3^, respectively. There was no statistically significant difference between them (P = 0.158). However, the median volume of residual RLNs after treatment in the ECCRT group was smaller than that in the CCRT group (0.58 cm^3^ *vs.* 2.59 cm^3^, P < 0.001). In addition, the median regression rate of RLNs was 81% in the ECCRT group, which was higher than 50% in the CCRT group (P < 0.001). (Table 3). And the median regression rate of RLNs was 66% in all patients.

**Table 2:** Baseline characteristics of the RLNs before radiotherapy

RLNs	ECCRT (n = 61)	CCRT (n = 61)	P
*Laterality*			1.000
Unilateral	37 (60.66%)	37 (60.66%)	
Bilateral	24 (39.34%)	24 (39.34%)	
*Necrosis*			0.091
Yes	27 (44.26%)	18 (29.51%)	
No	34 (55.74%)	43 (70.49%)	
*ENS*			0.361
Yes	29 (47.54%)	24 (39.34%)	
No	32 (52.46%)	37 (60.66%)	

*ENS* Extranodal neoplastic spread

**Table 3:** Regression rates of RLNs

	ECCRT Median (IQR)	CCRT Median (IQR)	P
*RLN*			
RNV_before_ (cm^3^)	3.90 (2.01,6.61)	4.81 (3.11,7.31)	0.158
RNV_after_ (cm^3^)	0.58 (0.32,0.96)	2.59 (1.34,3.75)	< 0.001*
Regression rates of RLNs (%)	81 (71.5,89)	50 (36.5,61)	< 0.001*

*IQR* interquartile range

*Statistically significant

### AEs

Neither cardiac dysfunction nor treatment-related bleeding occurred in either group. The acute AEs observed in both groups were myelosuppression, oral mucositis, skin reaction, nausea/vomiting, liver dysfunction, and renal dysfunction. The occurrence of grade 3/4 oral mucositis in the ECCRT group was 26.23%, which was lower than that (44.26%) in the CCRT group (P = 0.037). Meanwhile, there was no statistically significant difference in other AEs between the two groups. There was no patient discontinuing treatment due to AEs and none died from acute AEs.

The late toxicities included limitation of mouth opening, dysphagia, decreased vision, hearing loss, radiation-induced brain injury, and xerostomia. One grade 5 late AE was observed in the ECCRT group. The incidences of late AEs were similar between the two groups (P > 0.05) (Table 4).

**Table 4:** Treatment-related Aes

Toxicity	ECCRT group (n = 61)			CCRT group (n = 61)			P*
Acute AEs	Grade 0	Grade 1/2	Grade 3/4	Grade 0	Grade 1/2	Grade 3/4	
Leukopenia	22 (36.07%)	29 (47.54%)	10 (16.39%)	15 (24.59%)	37 (60.66%)	9 (14.75%)	0.803
Hemoglobin decrease	6 (9.84%)	48 (78.69%)	7 (11.48%)	12 (19.67%)	45 (73.77%)	4 (6.56%)	0.343
Liver dysfunction	53 (86.89%)	6 (9.84%)	2 (3.28%)	52 (85.25%)	8 (13.11%)	1 (1.64%)	1.000
Renal dysfunction	52 (85.25%)	9 (14.75%)	0 (0)	51 (83.61%)	10 (16.39%)	0(0)	-
Thrombocytopenia	32 (52.46%)	27 (44.26%)	2 (3.28%)	43 (70.49%)	17 (27.87%)	1 (1.64%)	1.000
Nausea/vomiting	30 (49.18%)	25 (40.98%)	6 (9.84%)	29 (47.54%)	29 (47.54%)	3 (4.92%)	0.491
Oral mucositis	5 (8.20%)	40 (65.57%)	16 (26.23%)	4 (6.56%)	30 (49.18%)	27 (44.26%)	0.037*
Skin reaction	16 (26.23%)	42 (68.85%)	3 (4.92%)	17 (27.87%)	39 (63.93%)	5 (8.20%)	0.717
Late AEs	Grade 0	Grade 1/2	Grade 3/4/5	Grade 0	Grade 1/2	Grade 3/4	P
Limitation of mouth opening	60 (98.36%)	1 (1.64%)	0 (0)	60 (98.36%)	1 (1.64%)	0 (0)	1.000
Dysphagia	60 (98.36%)	0 (0)	1 (1.64%)	59 (96.72%)	2 (3.28%)	0 (0)	0.573
Decreased vision	60 (98.36%)	1 (1.64%)	0 (0)	58 (95.08%)	3 (4.92%)	0 (0)	0.619
Hearing loss	55 (90.16%)	6 (9.84%)	0 (0)	53 (86.89%)	8 (13.11%)	0 (0)	0.570
Radiation-induced brain injury	59 (96.72%)	2 (3.28%)	0 (0)	60 (98.36%)	1 (1.64%)	0 (0)	1.000
Xerostomia	45 (73.77%)	16 (26.23%)	0 (0)	42 (68.85%)	19 (31.15%)	0 (0)	0.548

P* values showed the comparison results of grade 3/4 acute toxicity between the two groups

*Statistically significant

### Survival and prognosis analysis

**Fig. 1:**
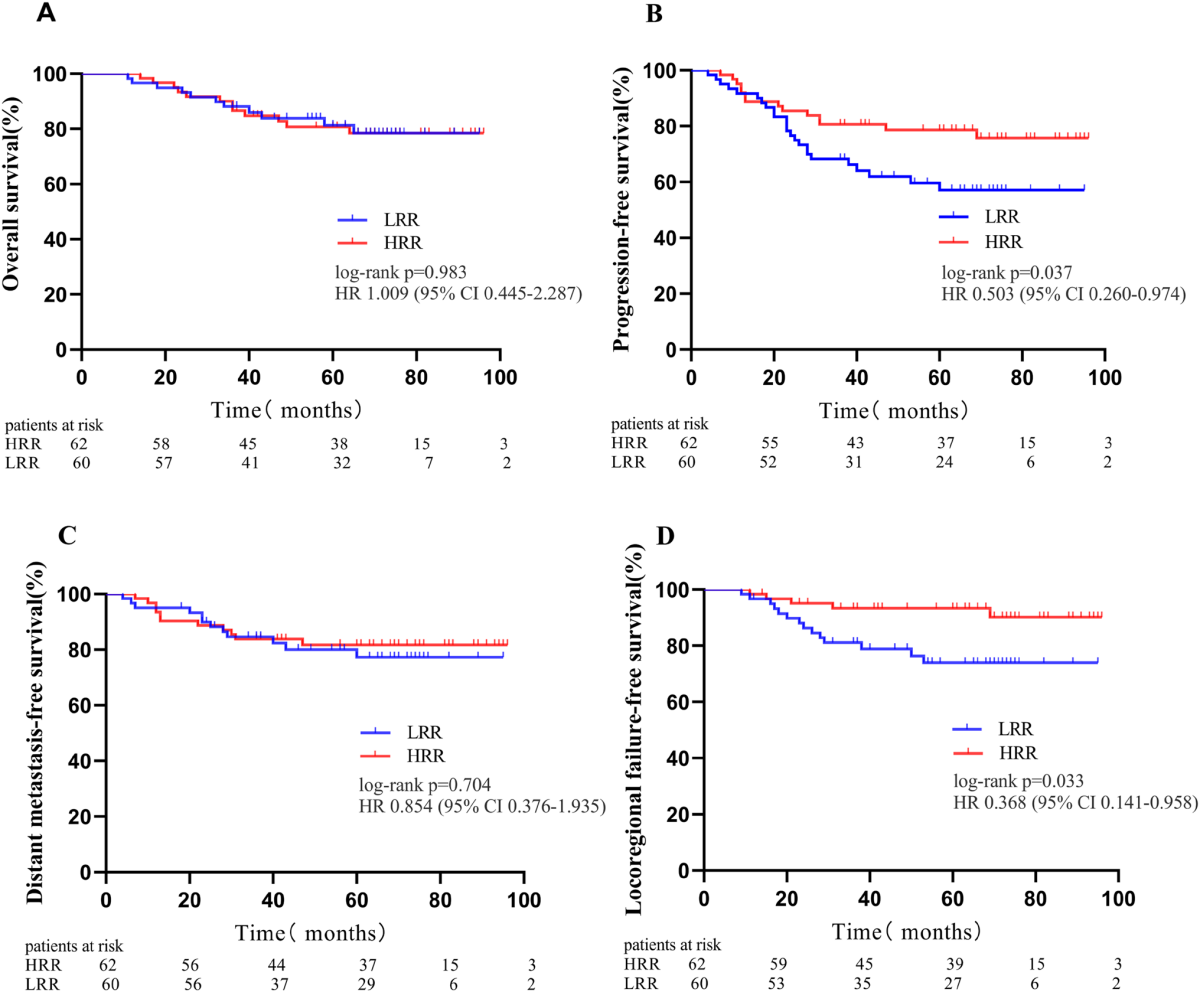
Kaplan -Meier survival curve of the different ratios of patients with LANPC in the HRR group and the LRR group. The 3-year overall survival rates (**A**), progression-free survival rates (**B**), distant metastasis-free survival rates (**C**), locoregional failure-free survival rates (**D**). *HR* hazard ratio, *CI* confidence interval

Patients were classified into two groups based on the median regression rate of RLNs: HRR (≥ 66%) and LRR (< 66%); The final follow-up time is May 15, 2023, with a median follow-up time of 65 months (range: 11–96 months). The 3-year OS, PFS, DMFS and LRFFS in the HRR and LRR group were 85.48% and 86.67% (HR 1.009, 95% CI 0.445–2.287, log-rank P = 0.983), 80.65% and 68.33% (HR 0.503, 95% CI 0.260–0.974, log-rank P = 0.037), 83.87% and 85% (HR 0.854, 95% CI 0.376–1.935, log-rank P = 0.704), 93.55% and 81.67% (HR 0.368, 95% CI 0.141–0.958, log-rank P = 0.033), respectively (Fig. 1). We also conducted univariable and multivariate analyses and found that regression rate of RLNs was an independent prognostic factor for PFS and LRFFS (Table 5). 12 patients (20%) died in the LRR group, of which 11 patients died of disease progression, 1 case died due to a car accident. In the HRR group, 14 patients (22.58%) died, of which 12 deaths were caused by disease progression, 1 patient died of second primary lung cancer, and 1 patient died due to dysphagia after radiotherapy. Disease progression occurred in 14 patients (22.58%) in the HRR group and 24 patients (40%) in the LRR group. Distant metastases were observed in 11 patients (17.74%) in the HRR group and 12 patients (20%) in the LRR group. In the HRR group, 6 patients (9.68%) had locoregional recurrence, while in the LRR group, 14 patients (23.33%) had locoregional recurrence.

**Table 5:** Multivariate analysis of factors associated with OS, PFS, DMFS, and LRRFS

Endpoint	Factors	HR	95% CI	P
OS	Gender (female vs male)	0.358	0.106–1.207	0.098
	Clinical stage (IVa vs III)	1.886	0.716–4.971	0.199
	Necrosis (yes vs no）	1.934	0.846–4.417	0.118
	N stage (N2 vs N0+1)	7.577	1.004–57.186	0.050
	N stage (N3 vs N0+1)	5.960	0.689–51.534	0.105
PFS	Group (HRR vs LRR)	0.482	0.245–0.947	0.034*
	Age (<60 vs ≥60)	0.392	0.168–0.910	0.029*
	N stage (N2 vs N0+1)	2.112	0.800–5.576	0.131
	N stage (N3 vs N0+1)	3.174	1.080–9.326	0.036*
DMFS	Laterality (bilateral vs unilateral)	1.758	0.762–4.054	0.186
	N stage (N2 vs N0+1)	7.505	0.983–57.279	0.052
	N stage (N3 vs N0+1)	9.704	1.165–80.812	0.036*
	Gender (female vs male)	0.361	0.107–1.217	0.100
LRRFS	Group (HRR vs LRR)	0.321	0.121–0.846	0.022*
	Laterality (bilateral vs unilateral)	0.280	0.092–0.851	0.025*
	N stage (N2 vs N0 + 1)	1.288	0.401–4.138	0.671
	N stage (N3 vs N0 + 1)	2.486	0.697–8.863	0.160

*Statistically significant

## Discussion

So far, numerous studies have focused on the treatment of cervical lymph nodes, but there is little research on RLNs. It has been reported that the incomplete regression of the primary tumor and/or metastatic lymph node at the end of radiotherapy was a predictor of poor outcomes in NPC patients (Liang et al. 2019). Bartelink’s study showed that tumors with a slow regression rate in head and neck squamous cell carcinoma had a high probability of recurrence (Bartelink et al. 1983). Moreover, previous research showed that the 5-year PFS, 5-year local recurrence-free survival (LRFS) rate in NPC patients with or without residual tumors after radiotherapy were 67.9% and 84.7% (P = 0.006), 80.4% and 93.4% (P = 0.002) (Lv et al. 2017). Li et al. reported that the 3-year PFS rates of patients with or without residual RLNs after treatment were 78.4% and 90.4% (P < 0.001), and the 3-year LRRFS rates were 93.3% and 96.9%, respectively (P < 0.001) (Li et al. 2020a, b). He et al. reported that the 3-year PFS rates of patients with or without residual tumors after treatment were 67% and 82% (P = 0.001), and the 3-year LRFS rates were 89% and 97%, respectively (P = 0.002) (He et al. 2015). Lee et al. pointed out that the regression rate of tumor is considered to have greater prognostic value than absolute tumor volume, and found that the tumor regression rate in patients without recurrence is higher than that in patients with recurrence (44.3% and 34.0%, p = 0.004), and patients with tumor regression rate greater than 35% have higher 5-year PFS than patients with tumor regression rate less than 35% (79.2% and 53.2%, P < 0.001) (Lee et al. 2016). Similarly in our study, patients in the HRR group have higher survival rates than those in the LRR group. The 3-year PFS rates in the HRR and LRR groups were 80.65% and 68.33% respectively (P = 0.037), and 3-year LRFFS rates were 93.55% and 81.67%, respectively (P = 0.033). Multivariate analyses found that regression rate of RLNs was an independent prognostic factor for PFS and LRFFS of LANPC patients. The 3-year PFS and LRFFS in our study were lower than those in the study of Li WZ, considering that our study only included patients with LANPC, while the study of Li WZ also included stage II patients, and the study of Li WZ was qualitative in RLNs, while our study was quantitative.

Due to the deep location and difficult dissection of RLNs in NPC, it is a difficult challenge to treat residual or recurrent RLNs. More than 50% of cases with secondary radiation therapy experienced severe radiation toxicity (Han et al. 2012). Therefore, how to improve the regression rate of RLNs is significant. In recent years, targeted therapy has become a promising anti-tumor treatment method. Researches have demonstrated that anti-angiogenic drugs and chemoradiotherapy have a synergistic effect on NPC (Lee et al. 2012; Zhang et al. 2018). Currently, the application of Endostar in NPC mainly focused on recurrent and metastatic patients (Guan et al. 2015; Jin et al. 2013), and had improved the complete remission rate of CLNs metastasis (Li et al. 2020a, b). However, it is unclear whether Endostar has an impact on the regression of RLNs, as both primary nasopharyngeal lesions and RLNs were previously unified as GTVnx in the radiotherapy target area. This is the first study to explore the role of Endostar in the regression of RLNs using accurate three-dimensional measurement of RLNs’ volume, which can provide some reference and new idea for the treatment of LANPC patients with RLNs metastasis. In this study, we separately outlined RLNs and found that the regression rate of RLNs in the ECCRT group was higher than that in the CCRT group (81% *vs.* 50%, P < 0.001).

Endostar has been shown to normalize blood vessels, thereby enhancing the blood supply to necrotic tissues and promoting tumor regression. Besides, it stimulates endogenous anti-angiogenic activity and inhibits VEGF activity, which results in a decrease in the number of microvessels, the oxygen consumption of immature blood vessels, and inflammatory exudation (Ling et al. 2007; Peng et al. 2012). According to the previous researches, Endostar can reduce radiation-induced tissue damage. It is reported that Endostar plus radiochemotherapy reduced the incidence of grade 3/4 oral mucositis compared to the group without Endostar (29.3% *vs.* 54.8%, P = 0.019) (Xu et al. 2022). Furthermore, Endostar reduced the occurrence of radiation-induced lung injury (Zhang et al. 2012), and the progression of early brain edema after radiation-induced brain injury (Ma et al. 2019). Analogously, Our study results indicated that patients in the ECCRT group exhibited a lower incidence of grade 3/4 oral mucositis compared to those in the CCRT group (26.23% *vs.* 44.26%, P = 0.037), which may be related to the radiological protective effect of Endostar on normal tissues.

## Conclusion

Our findings demonstrated that Endostar combined with CCRT is expected to become an effective and low-toxicity treatment method for LANPC patients with RLNs. Moreover, the high or low RLNs regression rate can help clinical doctors develop personalized treatment strategies and provide intensive treatment for patients with poor RLNs regression. However, there are some limitations to our study. Firstly, this is a retrospective cohort study. We cannot completely alleviate selection bias. Secondly, positive RLNs diagnosis relied on MRI due to the difficulty in obtaining pathological tissue. Thirdly, this is a single-center study that lacks external validation from other centers.

## Data Availability

The data underlying this article will be shared on reasonable request to the corresponding author.
